# Application of InSAR and GIS Techniques to Ground Subsidence Assessment in the Nobi Plain, Central Japan

**DOI:** 10.3390/s140100492

**Published:** 2013-12-31

**Authors:** Minxue Zheng, Kaoru Fukuyama, Kazadi Sanga-Ngoie

**Affiliations:** 1 Graduate School of Bioresources, Mie University, Tsu, Mie 514-8507, Japan; E-Mails: zmx503d707@yahoo.co.jp (M.Z.); fukuyama@bio.mie-u.ac.jp (K.F.); 2 Laboratory of Environmental Geoscience, Ritsumeikan Asia Pacific University, Beppu, Oita 874-8577, Japan

**Keywords:** ground subsidence, satellite InSAR, GIS, atmospheric path delay, Analogous weather charts method, Nobi Plain

## Abstract

Spatial variation and temporal changes in ground subsidence over the Nobi Plain, Central Japan, are assessed using GIS techniques and ground level measurements data taken over this area since the 1970s. Notwithstanding the general slowing trend observed in ground subsidence over the plains, we have detected ground rise at some locations, more likely due to the ground expansion because of recovering groundwater levels and the tilting of the Nobi land mass. The problem of non-availability of upper-air meteorological information, especially the 3-dimensional water vapor distribution, during the JERS-1 observational period (1992–1998) was solved by applying the AWC (analog weather charts) method onto the high-precision GPV-MSM (Grid Point Value of Meso-Scale Model) water-vapor data to find the latter's matching meteorological data. From the selected JERS-1 interferometry pair and the matching GPV-MSM meteorological data, the atmospheric path delay generated by water vapor inhomogeneity was then quantitatively evaluated. A highly uniform spatial distribution of the atmospheric delay, with a maximum deviation of approximately 38 mm in its horizontal distribution was found over the Plain. This confirms the effectiveness of using GPV-MSM data for SAR differential interferometric analysis, and sheds thus some new light on the possibility of improving InSAR analysis results for land subsidence applications.

## 1. Introduction

Except for the hilly Yoro Mountains along its western border, the Nobi Plain in Central Japan is the largest flat area in Japan, covering about 1,800 km^2^. Ground subsidence is the most remarkable ground surface process observed over this area. It is known to encompass the biggest large-scale ground-subsidence zone in Japan, centered around Nagoya City and the Ise Bay. The total subsidence area covers approximately 740 km^2^: this is more than one third of the whole of the Nobi Plain.

Ground subsidence over the Nobi Plain is a natural phenomenon occurring due to natural compaction of the soft sedimentary layers of the plain and the tilting of the Nobi geomorphologic structure itself [1–3]. Subsidence rate of 23 cm/year was recorded at an observational point in Minato Ward, Nagoya, in 1973. However, such a large-scale subsidence cannot be explained by the above-mentioned factors alone. The acceleration of compaction and contraction due to falling groundwater levels can be pointed as one of the other major man-made factors with potentially remarkable impacts. These falling levels are a consequence of the fast increase in pumping ground water following the rapid postwar economic growth over the area, and which could not be matched by natural replenishment of ground waters [1–4]. Similar ground level subsidences have also been reported in relationship with the overexploitation of ground waters in many other parts of the World (see [5] for a detailed review).

This ground subsidence over the Nobi Plain became well known following the Ise-wan Typhoon (Typhoon Vera) in autumn 1959. In its Annual Report in 2000, the Land Subsidence Survey Committee of the Three Prefectures in Tokai Region reported that, after the 1959 typhoon, a gradual expansion of the subsidence area peaking in 1973–1974 was observed, followed by a slowing trend in subsidence activity since then, more likely because of such factors as the strengthening of regulations concerning groundwater pumping [6]. We have to note here that very few researches have been performed on this subject so far, notwithstanding the remarkable ground subsidence still observed to occur in some areas of this Plain, even nowadays, or the rising grounds found in many other parts. These changes in ground level conditions are likely to imperil the foundations of life-sustaining infrastructures in this area, including damages to built up structures, especially in the case of tsunamis or sea level increase due to the global warming. All these facts are a great source of concern for residents in the region [7].

Ground leveling, the traditional method for observing ground subsidence, makes it possible to directly measure ground subsidence with quite high precision. However, this method remains crippled with many issues, among which the shortage in manpower, the volume of labor and the cost required for performing the measurements, the cost for the maintenance and management of observational points, as well as the inability to get observation information for those areas not included in the leveling routes and ground points network [8,9].

The analysis on ground changes by InSAR can be expected as in recent years, interferometric synthetic aperture radar (InSAR) technology [10–12] has been more and more in use to estimate with high precision the spatial distribution of changes in the Earth's crust surface height and the amount of such changes at each specific location [13–19]. This new approach can be considered as a complementary method to by traditional standard measurements for monitoring ground subsidence. More specifically, ground level subsidence reported in relationship with the overexploitation of ground waters has been analyzed using InSAR techniques in many parts of the World. This is the case of Lisbon in Portugal [20], the Pingtung Plain [11] and the Chousui River Alluvial Fan [21] in Taiwan, the Campania Region [22] and Bologna [23] in Italy, as well as Kolkata City in India [24]. It is expected the InSAR techniques, as well as its more recent variants such as the PSInSAR [25], will become powerful complementary, or even substitute, methods to traditional ground subsidence observation using leveling and other methodologies used so far.

In these perspectives, the Nobi Plain can be considered as a promising area for developing new methodologies aiming at ground subsidence research using satellite InSAR data. However, during their work on estimating ground subsidence from satellite data, Fujiwara *et al.* [26] and Shimada [27] noted that it was effectively quite difficult to differentiate the atmospheric delay phase due to water vapor from the phase due to changes in the Earth's crust. The atmospheric delay itself is found to be a direct cause of remarkable observational error. For this reason, in order to increase the precision of change detection in the Earth's crust, research on the effects of water vapor on microwaves, and their mitigation, is essential [5,27–30].

Changes in atmospheric water vapor are extremely complex, with 3-dimensional changes taking place not only in the vertical, but also in the horizontal directions [5,29,31]. It is extremely difficult to correct for the local effects of water vapor when local aerological data are not available [32]. The PSInSAR method attempts to handle this issues by temporal averaging of up to 30 SAR images [9]. Conventional researches with respect to the atmospheric impacts on InSAR mainly examine the effects of changes in atmospheric water vapor with altitude, limiting therefore the precision of this methodology in most of the past studies on atmospheric delay using InSAR techniques [33,34].

There is therefore a great urge nowadays for quantitatively obtaining 3-dimensional spatial distributions of water vapor in order to estimate the atmospheric delay on InSAR data, as due to the changes in atmospheric water vapor contents. This is a very challenging issue, especially when one has to quantitatively assess these distributions and the related atmospheric impacts in order to use them with the JERS-1 SAR data. We have to note here that these satellite data were observed from 1992.01.11 to 1998.10.12, a period when no archived upper-level meteorological data are available over Japan.

In this study, using GIS as analytical platform, we aim at estimating the spatial variation and the temporal changes in ground subsidence over the Nobi Plain using both ground level measurements data and InSAR data. However, notwithstanding the availability of weather charts and detailed information of ground surface atmospheric conditions (temperature, pressure, water vapor, wind, *etc.*) over Japan during the JERS-1 period (1992–1998), detailed information for upper atmospheric layers has been made available by the Japan Meteorological Agency only after 2002 with its multi-layer and multi-temporal Grid Point Value of Meso-Scale Model (GPV-MSM) data set (see Section 2.2 for details). We therefore propose to use the Analog Weather Chart (hereafter, AWC) method [35,36] in order to estimate from the analog GPV-MSM weather charts and datasets those water vapor inputs needed for calculating water vapor effects on the JERS-1 SAR interferometry data.

In our analysis, quantitative estimation and assessment of the impact of water vapor on InSAR data are performed, especially in terms of atmospheric delay generated by tropospheric water vapor inhomogeneity. The AWC method is used to provide 3-dimensional atmospheric information from the GPV-MSM data matching those for the days of the selected JERS-1 interferometry pairs over the Nobi Plain, days when detailed upper level meteorological data are available. In the following, data sets and the analysis method are described in Section 2, research results and discussions are given in Sections 3 and 4, respectively, followed by concluding remarks in Section 5.

## 2. Data Sets and Analysis Method

### 2.1. Ground Level Data

**Figure 1.**
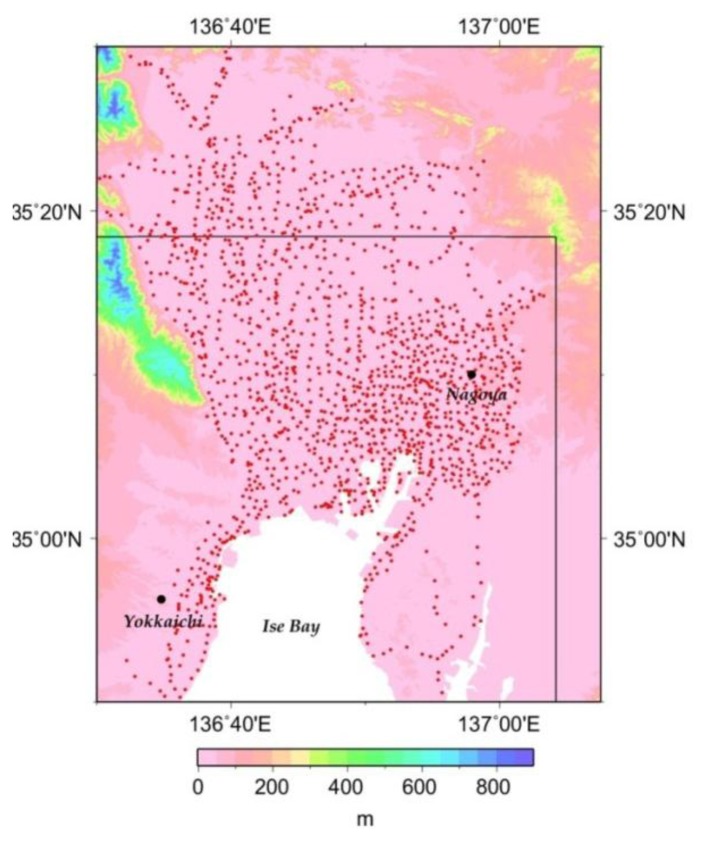
Distribution of leveling points (pink dots) over the Nobi Plain, around Nagoya. The solid line denotes the area of study in this work. Altitude (in m) is shown by the legend code.

For our ground subsidence analyses, we use ground level data from surveys conducted on annual basis over the Nobi Plain since 1971 by the Land Subsidence Survey Committees of the three Prefectures (Aichi, Gifu and Mie) in the Tokai Region, at approximately 1,500 sites in total (Figure 1).

Data processing involved, first of all, the verification of erroneous data. Using the temporal time series of observational data at each observational point, we calculated the standard deviation, and data with too large errors (outliers) were dropped from further calculations as erroneous. These outliers could be understood either as resulting from the changes in the location of the observational points, or as observational errors or errors/mistakes that occurred during data transcription. After this verification and correction, a database of the changes in ground elevation was prepared by converting the observed point data into a raster spatial database using GRASS, the world-known open access GIS software.

### 2.2. GPV-MSM Mesoscale Data

In Japan, research conducted on atmospheric delay by water vapor prior to 2003 used mainly the Japan Meteorological Agency's Global Analysis (GANAL) data. Since July 2002, the Japan Meteorological Agency (JMA) has been releasing every day GPV-MSM data in the form of 18-hour forecast data with initial values at the following four moments: 03:00, 09:00, 15:00, and 21:00. These data consist of ground-level and upper-atmosphere aerological data described as follows.

The ground-level data, prepared for every hour, consist of the data measured at every 0.1 degrees latitude and 0.125 degrees longitude (approximately 10 km) gridded points for the following physical parameters: sea-level air pressure (hPa), wind direction (rad), air temperature (K), relative humidity (%), precipitation (mm), and upper-, mid-, and lower-atmosphere cloud cover. The upper-atmosphere aerologic data are isobaric data measured every three hours at 14 layers (from the 975 hPa to the 100 hPa isobaric levels) at gridded points separated by 0.2 degrees latitude and 0.25 degrees longitude (approximately 20 km) for the following atmospheric parameters: geopotential height (m), wind direction (rad), and air temperature (K). Relative humidity (%) and vertical wind component (hPa/h) data are available for the 10 standard layers up to 300 hPa. Hereafter, atmospheric delay will be quantitatively estimated based on the difference in water vapor distribution from weather charts matching by the AWC Method for JERS-1 interferometry pairs in the Nobi Plain.

### 2.3. The Analog Weather Charts (AWC) Method

While atmospheric conditions do naturally vary between different locations on the earth, they also vary from year to year over the same location. Ordinarily, such variation remains within a certain range from year to year, repeating itself on an annual cycle. Climate refers to the most probable general atmospheric long term average conditions repeating from year to year over a given location, basically in accordance with this one-year cycle [37]. The pattern-matching method for weather charts (also known in the literature as the “Analog Weather Chart Method”) is a method currently used for weather forecasting, which consists of identifying a past weather chart that closely resembles to, or matches, the current one and then using its later changes to forecast future weathers [38–40]. World leading Meteorological Services (in USA, Germany, France, England, Sweden) have been using this method for extended range forecasts, especially since the early 1940s [35,36,38–43]. This has also been adopted since by many other countries all over the world.

Using this method, we first identify weather charts from any day in 2003 or later (for which GPV-MSM data are available) closely resembling the weather chart for the date for which JERS-1 data were obtained (inputs “X”), and use them as “Y” inputs. In order to find similar weather charts, we compare not only the spatial distribution of isobars and the position of the rain fronts, but also the values and locations of the high and lower pressure fields relative to the observation points. When the match is found, we assume that weather conditions were similar on these two days (X and Y), and secure the GPV-MSM data (day Y) as equivalent to those of the day X of JERS-1 Atmospheric delay is then estimated using these data, and the impact of water vapor distribution on the satellite InSAR measurements is thus assessed.

In this study, the following conditions have been added to this methodology in order to increase the precision of performance in identifying similarities between weather charts for the different two analysis days: (1) the X and Y days must belong to the same season; (2) the climatic parameters observed on the earth's surface in the study areas must be similar, and (3) aerological data measured over the areas of concern must be similar. With this, we ascertain that GPV-MSM data satisfying these conditions are the most likely to reflect the weather conditions on the date of collection of JERS-1 data.

### 2.4. Selection of GPV-MSM Data

The selection of GPV-MSM data was made through the following five steps: (1) preparation of a surface weather chart contemporaneous to the JERS-1 observation date (X); (2) identification of a similar weather chart for the year 2003 or later (Y); (3) comparison of surface weather data (sea-level air pressure, air temperature, water-vapor pressure) for Nagoya on both X and Y days; (4) comparison of aerological meteorological data (geopotential height air temperature, water-vapor pressure) on the 925 hPa, 850 hPa, and 800 hPa isobars on the date Y with radiosonde aerological data at Shionomisaki (the closest radiosonde station to Nagoya) on the observation date (X); and (5) obtaining GPV-MSM data for the most analog date Y. In practice, during step 5, the weather charts, the surface weather data, and the radiosonde aerological isobar weather data, obtained from steps 1 through 4, were subjected to a comprehensive and in-depth examination.

### 2.5. SAR Interferometry and Atmospheric Path Delay

In this study, we used JERS-1 SAR data (L-band: 23.6 cm; HH polarization; Swath width: 75 km; Resolution: 18 m) [10,11] observed over the Nobi Plain on 6 May and 19 June 1998 for estimating the atmospheric delay, and processed the data using the SIGMA-SAR processor [27] in order to obtain the differential interference image from which phase features could be deduced.

Shimada (1999) [27] proposed a method for correcting the atmospheric path delay from global analysis data using the following equations:

(1) $$\varphi =-4\pi \frac{2}{\lambda_{0}\sin2\Theta_{0}}\sum_{i}\left(n_{m,i}-n_{s,i}\right)\Delta r_{i}$$

(2) $$n=1+\frac{77.6}{T}p\cdot 10^{-6}+\frac{0.373}{T^{2}}e$$

where, *Θ_0_* represents the off-nadir angle at the satellite point, *n*_m,i_ and *n*_s,i_ are the indices of refraction for the atmospheric layer *i* of the master and slave satellite images, respectively, and Δr_i_ the thickness of layer i. In the empirical formulation for the index of refraction (Equation (2)), *p*, *T*, and *e* represent the atmospheric pressure (hPa), the air temperature (K), and the water-vapor pressure (hPa), respectively. Some other variants of this basic formulation can be found in the literature [12,30]. We have opted to use Equations (1) and (2) in this paper for estimating the atmospheric path delay using GPV-MSM data, not only because of their respective computational simplicity, but mostly because of the fact that they include the dependency of the refractive index *n* in terms of both the pressure (*p*), therefore the altitude (hydrostatic delay), and the water vapor pressure (*e*) (wet delay) that is known to strongly vary horizontally [30]. The deviation in atmospheric delay from the mean value over the area of investigation is then calculated with 0 m above sea level as reference surface.

## 3. Results

### 3.1. Ground Subsidence

**Figure 2.**
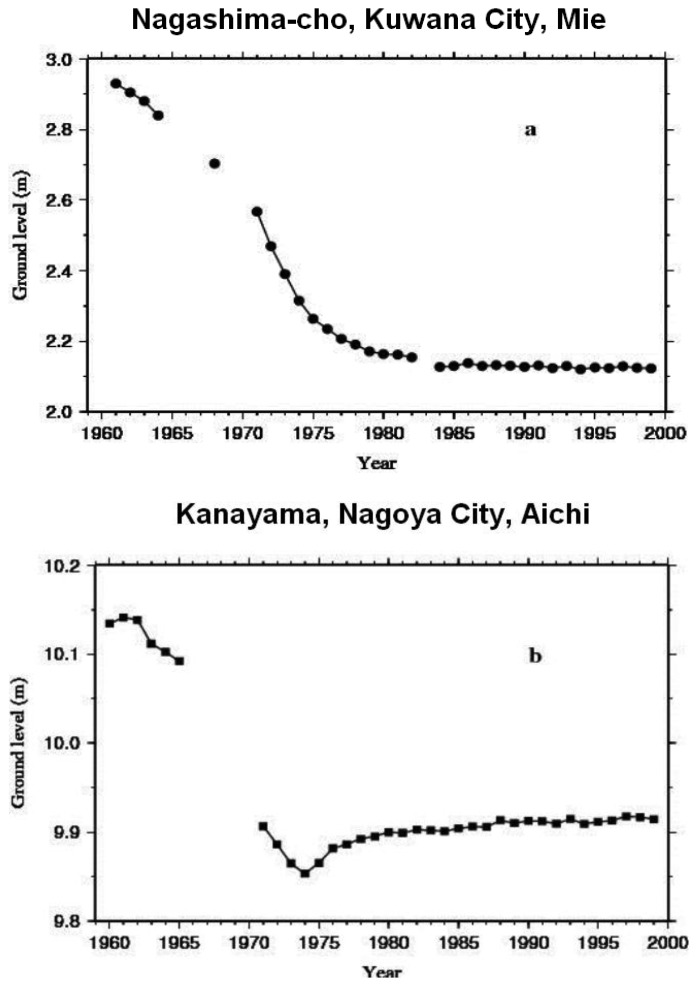
Observed ground level (in mm) changes over the Nobi Plain, at (**a**) Kuwana and (**b**) Kanayama. Discontinuities in the observational points denote missing data.

From the time-series data at each observational point (Figure 1), we deduced that the observational period could broadly be categorized into two separate trends, marked by a rapid subsidence up to the early half of the 1970s, followed by a slower trend noticeable in the second half of the 1970s (Figure 2a,b). Observational points (Figure 1) can thus be divided into two groups: those where subsidence had continued after 1980s (Figure 2a: Kuwana), and those where a gradual rising has been observed since then (Figure 2b: Kanayama). An increasing trend in the number of points with gradual rising was also noticed. It is conceivable that this rising in ground levels could be the result of ground expansion due to groundwater levels recovery following, among others, the decreased pumping of groundwater relative to such factors as countermeasures implemented by local and national government agencies, structural industrial changes and plate tectonics in connection with the tilting of the Nobi Plain.

**Figure 3.**
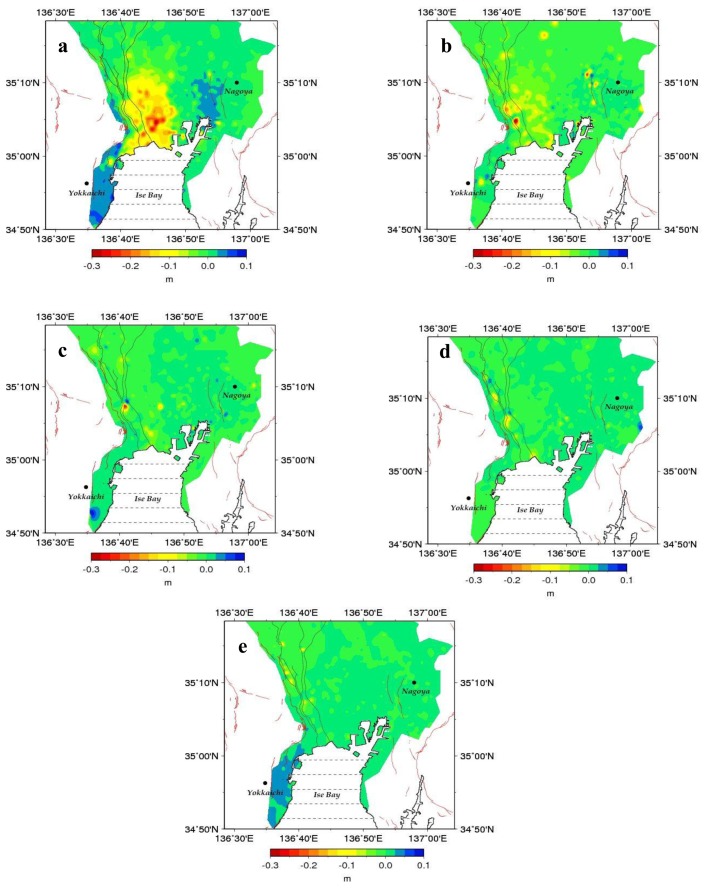
Variation in ground elevation (in m) using leveling measurements data. (**a**) 1975–1979 period. (**b**) 1980–1984 period. (**c**) 1985–1989 period. (**d**) 1990–1993 period. (**e**) 1994–1998 period.

From our spatial analysis results including frequency distributions of the changes in ground elevation, isopleth change diagrams, and time-series data for each observation point, it became clear that, up to the 1980s, ground subsidence occurred over a wide area of the Nobi Plain, with remarkably high amounts of subsidence at the mouths of the wide estuaries of the three main rivers of the Kiso group (Kiso, Nagara and Ibi Rivers) and that of the Shonai River, especially in the vicinity of the Nagoya Port (Figure 3a).

While ground subsidence has eased over most of the area since the 1980s and a gentle rising of the ground has begun in the area south of the Yoro Fault and elsewhere, continued subsidence is still observed over the area from the estuaries of the Kiso group of Rivers to the Waju district (Figure 3b,c). This rising trend is still observable during the 1990s, even in such urban areas as Nagoya, more likely in response to the previous severe subsidence that had induced relatively large amounts of land deformation to spread over unto the vicinity of those loci which were affected the most by subsidence (Figure 3d,e).

**Figure 4.**
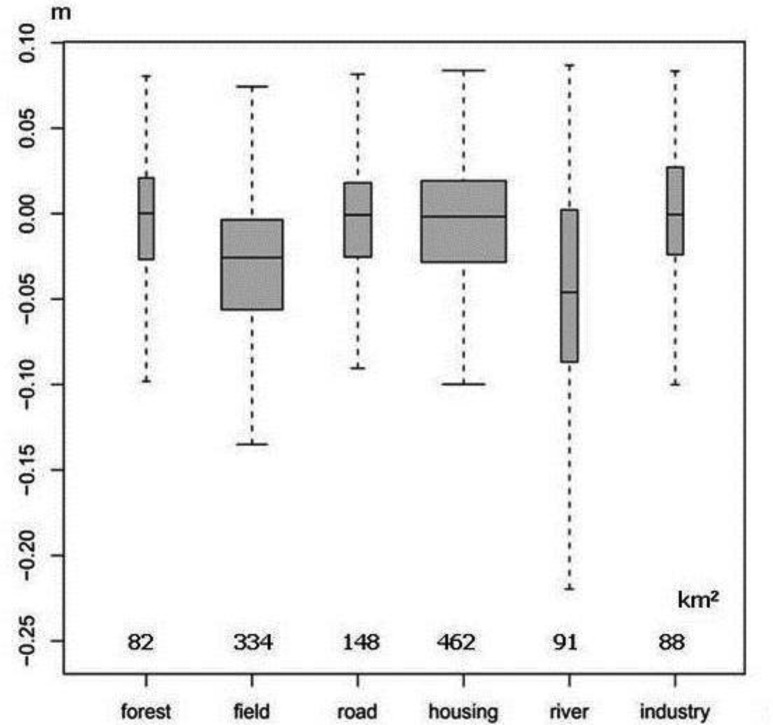
Variation in ground elevation by land use.

A relationship between land use and land cover (LU/LC) categories in the Nobi Plain and the amount of variation in land elevation is highlighted: the amount of land subsidence tends to be larger in areas covered by water bodies and smaller on industrial lands (Figure 4). In general, most of firm foundation structures are found on industrial lands. The rate of subsidence by these structures is seen to be strongly reduced, more likely because they are anchored in a more secured manner to the deep bedrock. Although the subsidence effects are felt across the area as a whole, this anchoring makes industrial structures less affected by such factors as land compaction due to groundwater removal or land expansion consecutive to groundwater recovery [44].

### 3.2. Selection of Analog Weather Charts

In order to identify analog weather charts, (step 2 of our methodology, see Section 2.4), we mainly compared the spatial distribution of isobars and the positions of seasonal rain fronts on the charts. Water vapor contained in the atmosphere is limited by temperature, and there is a maximum level that cannot be exceeded. While comparing the meteorological elements (steps 3 and 4 of our methodology), it became more apparent that air temperature was the most influential element for our analysis. In fact, when comparing surface observation data in step 3, we set empirical threshold values of 5 °C for the difference in air temperature and 5 hPa for the difference in sea-level air pressure.

**Figure 5.**
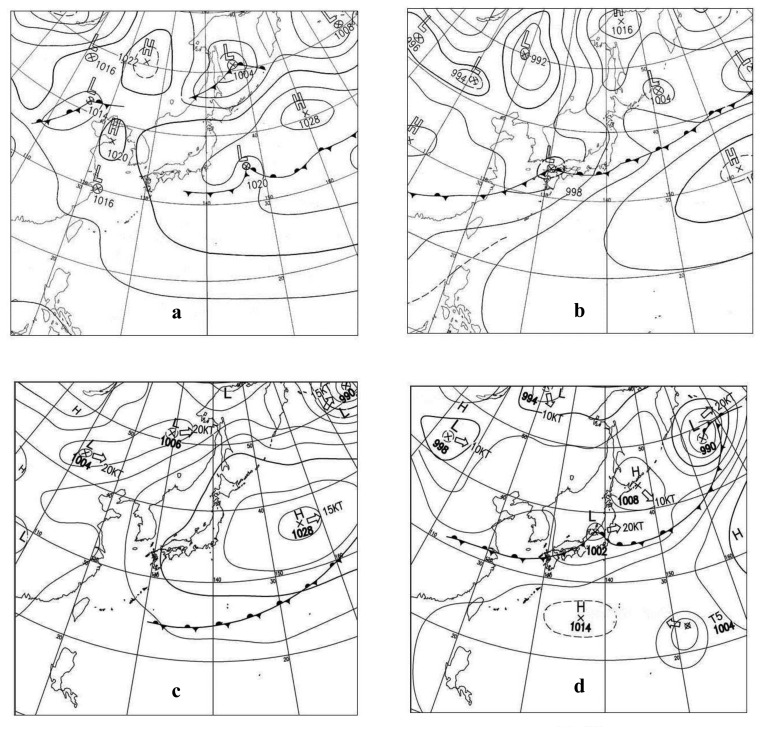
Weather chart. (**a**) 6 May 1998. (**b**) 19 June 1998. (**c**) 27 May 2004: the date with weather conditions most analogous to those of 6 May1998. (**d**) 12 July 2005: the date with weather conditions most analogous to those of 19 June 1998. Wind speed is expressed in knots (KT) and wind direction by thick open arrows, while values of the pressure at the center of the High (H) or Low (L) cores marked by a “×” are given in millibars (mb).

JERS-1 images captured on 6 May and 19 June 1998 were selected as interference pair for the analysis. For this purpose, using the AWC Method (see [33] for details), comparison of GPV-MSM aerological observation data (step 4) shows similarity in meteorological elements of the 6 May 1998, weather map and those of 2 May and 27 May 2004 weather maps. Similarly, weather conditions on 26 June 2004 matched those of 19 June 1998 and 12 July 2005. Referring to wind direction and wind speed data from surface weather data observed at Yokkaichi and radiosonde aerological observation data from Hamamatsu, we finally selected 27 May 2004, and 12 July 2005, as the dates with weather conditions the most similar to those of 6 May 1998, and 19 June 1998, respectively (Figure 5). The GPV-MSM upper data of these selected two days were used later on for the calculations.

### 3.3. InSAR Atmospheric Path Delay

The weather on JERS-1 SAR observation dates was cloudy on 6 May and raining on 19 June 1998. The spatial distribution of water vapor on both dates was not uniform, and it is possible that atmospheric delay caused by the water vapor distribution might have had a major impact on the differential interference phases. The JERS-1 SAR data observed over the Nobi Plain for these two dates were analyzed for the estimation of the atmospheric path delay using the SIGMA-SAR processor [26,44] according to the method described in Section 2.3.

**Figure 6.**
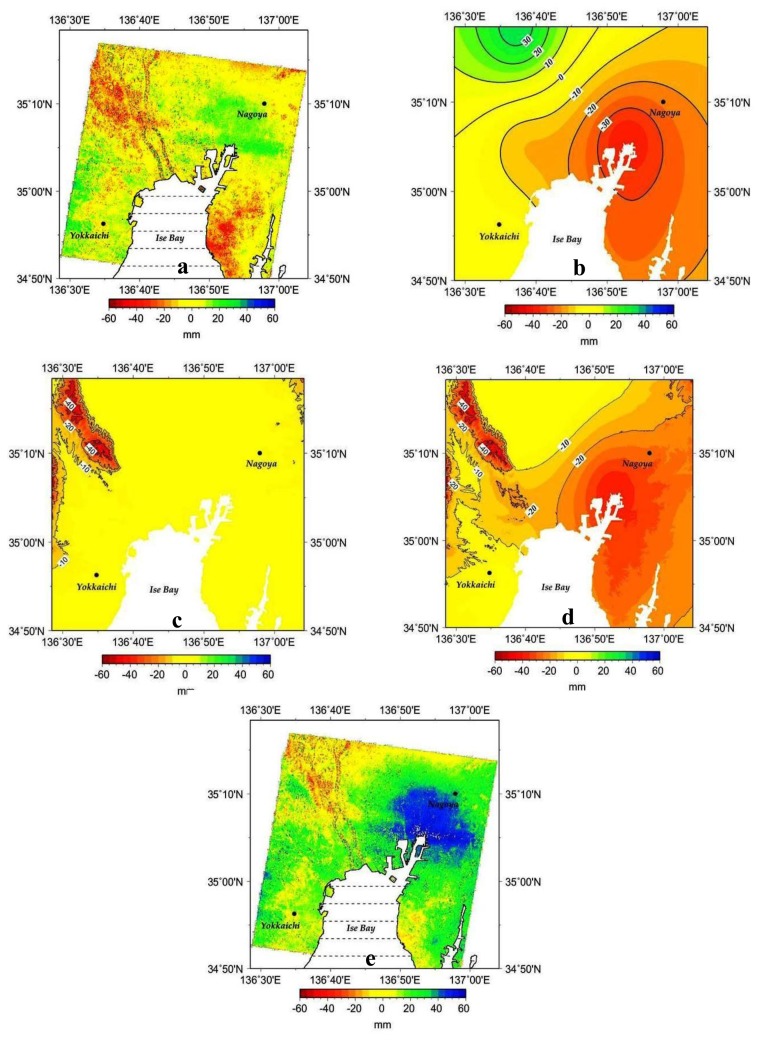
Ground subsidence analysis results. (**a**) Land surface deformation map calculated from the differential interferometry of the JERS-1 SAR observation pair (6 May and 19 June 1998) over the Nobi Plain. (**b**) Land surface deformation due to the atmospheric delay induced by the horizontal distribution of atmospheric water vapor. (**c**) Same as in (b), but for the atmospheric delay induced by topography (vertical distribution of water vapor). (**d**) Same as in (b), but for the atmospheric delay due to the combined horizontal and vertical effects of water vapor. (**e**) Corrected land deformation by considering the atmospheric delay induced by the horizontal and vertical distribution of water vapor.

The land surface deformation map obtained from differential interferometric analysis of the JERS-1 pair over the Nobi Plain (Figure 6a) shows substantial ground level changes over the northwest and southeast areas, with an increase in the direction of the line-of-sight. The deviation map (in mm) due to atmospheric delay induced by the changes in the horizontal distribution of water vapor obtained from the selected pair of dates (27 May 2004 as the most similar date to 6 May 1998, and 12 July 2005 as the most similar date to 19 June 1998) is given in Figure 6b. Major changes can be seen in the southeast region, with the greatest deviation value of approximately 38 mm.

Figure 6c shows the results deformation related to the atmospheric delay induced by topography in the SAR observation direction, as obtained by using the 50 m-grid digital elevation model (DEM) provided by the Geospatial Information Authority of Japan, and the GPV-MSM data. This effect is remarkable only in the northwest region. The spatial distribution of the combined effects of the atmospheric delay obtained from changes in water vapor in the horizontal direction (Figure 6b) and that from the topography (vertical direction, Figure 6c) is shown in Figure 6d. Major changes in the surface elevation map are found in the northwest and the southeast (centered on the Nagoya Metropolis) of the area of our analysis.

### 3.4. Corrected Differential Interference Image Considering the Atmospheric Delay

The corrected differential interference image between the 19 June and 6 May 1998 pair is given in Figure 6e. This correction is performed by considering the atmospheric path delays calculated from both the changes in water vapor in the horizontal direction (Figure 6b) and that from topographic effects (Figure 6c). Compared to Figure 6a, the corrected distribution in Figure 6e is characterized by major decreases in surface elevation values in the northwest and the southeast. Some residual changes can still be observed in the northwestern area, more likely as a signature of the presence of some undetected ground subsidence.

## 4. Discussion

The results showed that ground subsidence is clearly in a slowing trend over the plains. Some locations are observed to have some ground rise, more likely due to the ground expansion because of recovering groundwater levels and because of the tilting of the Nobi land mass. We have to note, however, that, the Nobi Plain being an area of extended ground subsidence, the underground structure in this region might not yet have reached the final stage of compaction. Our results compare to those of many other parts of the world, characterized by excessive groundwater pumping for big megalopolis built over alluvial lands [20–24]. It can be said that, notwithstanding the present trend of gently rising grounds, it is still more likely that, in the case of such actions as the resumption of excessive pumping of groundwater, ground subsidence could occur again, and on the large-scale.

Because of preset times at which satellite SAR data and GPV-MSM data are obtained, collecting data from different platforms on the same location and date will necessary involve the acceptance of a given time difference in data capture. Knowing that JERS-1 SAR data are obtained over Japan by around 10:30 am, we opted to use in our analysis initial GPV-MSM data from the nearest point in time, *i.e.*, 9:00 am, with a data capture difference of approximately 90 min.

In most of the previous studies, it was assumed that there was no change in the weather conditions related to this difference in times and that the physical quantities in the atmosphere were similar at the different times [27,45,46]. Recent interferogram stacking methodologies, such as the PSInSAR, try to get around this problem by averaging multi-temporal SAR images, but we cannot ascertain that the atmospheric water vapor issue is thus effectively solved.

In the present study, using the AWC Method, we identified dates with the most similar weather conditions in GPV-MSM data as compared to those of the JERS-1 observation dates, and used such important parameters as the air pressure, temperature, water-vapor pressure, and other meteorological elements that influence the index of refraction in the atmosphere. For this reason, even though there is a difference of approximately five years between the times at which GPV-MSM data and JERS-1 SAR data were collected, the GPV-MSM data could reflect high similarity with the weather conditions on the dates when the JERS-1 SAR data were collected. Further research is still needed to improve the precision of this pattern-matching scheme used in our Analog Weather Charts method, especially by using clear-sky data only for the analysis.

The maximum deviation obtained due to the atmospheric delay relative to the horizontal distribution of water vapor over the studied area is approximately 38 mm, with the highest deviation values over the southeast area (Figure 6b). The deviation induced by topography is shown to occur mostly over the northwestern region of the studied area, and is estimated to be approximately 53 mm in mountainous terrain 898 m above sea level, when the phase base surface is set to 0 m above sea level (Figure 6c). There is acceptable similarity between the spatial distribution patterns of changes based on the atmospheric delay derived by applying GPV-MSM data (Figure 6d) and those based on SAR interferograms (Figure 6a). For this reason, we would suggest that the changes detected in the northwest of Figure 6a are more likely due to atmospheric delay from topography-related water vapor changes, while those in the southeast underline atmospheric delay accompanying horizontal changes in atmospheric water vapor.

**Figure 7.**
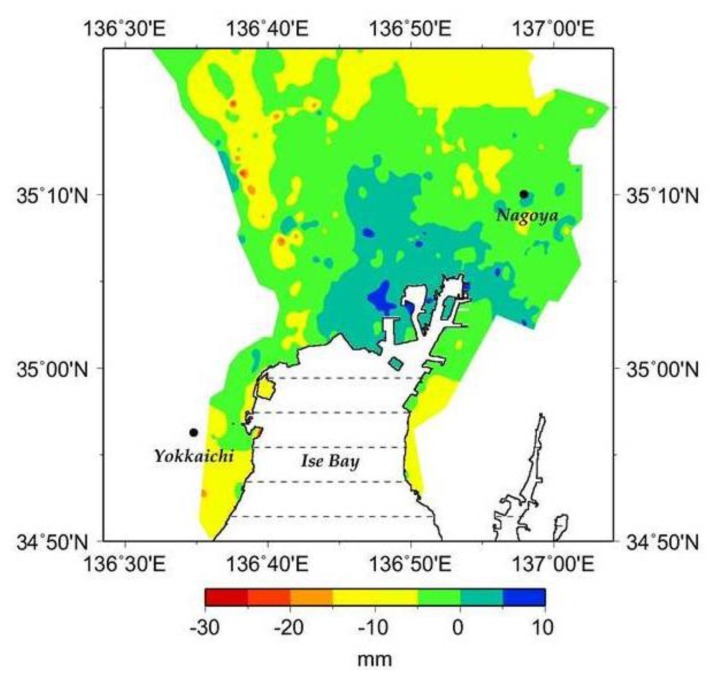
Variation in ground elevation using 1998 leveling measurements data.

The present work has shed some new light on the possibility for correcting quantitatively the interferometric data by using atmospheric delay estimated from water vapor data selected from GPV-MSM data records by the AWC metodology. However some phase residuals can be noticed in the northwestern area, more likely because of either one, or all, of the following three possible reasons: (1) occurrence of ground subsidence over this area as shown for 1998 in Figure 7; (2) the difference in the values of the meteorological elements between the JERS-1 observation dates and the identified dates for GPV-MSM data, notwithstanding the similarity of the data at the two dates; (3) errors due to the coarse resolution of the GPV-MSM data as compared to the JERS-1 SAR data.

Recently, the Japan Meteorological Agency has been providing GPV-MSM data with 5 km-grid surface measurements and 10 km-grid aerological isobar measurements. We foresee that the use of these fine-mesh GPV-MSM data in the future will contribute to increasing the precision in estimating the effects of water-vapor distribution on atmospheric phases delay, and therefore, to improving land subsidence analyses using InSAR data.

## 5. Conclusions

In this study, we were able to encode geographically the positions of land subsidence observational points over the Nobi Plains and to analyze such issues as the direct relationships between the changes in ground elevation over the years and the land use/land cover features using ground features raster data prepared on a GIS platform. Spatial distribution of ground subsidence shows a high level of subsidence near the mouths of the Kiso Three Rivers in particular.

Moreover, we made it possible to assess the effects of atmospheric water-vapor distribution on SAR satellite observation data using high-precision GPV-MSM water-vapor distribution data for those SAR observation dates when detailed upper-layers meteorological data are not available. Using a formulation that includes the dependency of the refractive index *n* on both the pressure (*p*), therefore the altitude (hydrostatic delay), and the water vapor pressure (*q*) (wet delay) that is known to strongly vary horizontally [26], we were able to assess the spatial distribution of atmospheric delay, and therefore, the role of the water vapor pressure field on SAR microwaves.

Our results show that the distribution of land deformation based on the atmospheric delay estimated from GPV-MSM water-vapor data is highly uniform spatially, with a maximum value of 38 mm over the study area. Similar results are obtained for the spatial distribution patterns of land deformation based on atmospheric delays estimated by using GPV-MSM data and differential interferometric SAR phases data, confirming therefore the effectiveness of using GPV-MSM data for SAR interferometric analysis.

The methodology developed in this work clearly shows the possibility of comparing estimates of ground subsidence made by using traditional ground surveys, with those derived from the analysis of InSAR data. Using finer mesh GPV-MSM data will thus more likely lead not only to higher precision in the estimates of changes in ground level by using InSAR techniques, but also to finer resolution ground level estimates as well. Comparing the present findings with those obtained by using clear-sky GPV-MSM data for the analysis is another avenue to be explored. Noting that InSAR analysis makes it possible to estimate ground subsidence by remote sensing even for those areas where ground survey or any other data collection methods are not possible, we foresee that the combination of these remotely sensed analysis data with those obtained by traditional leveling methods opens the way to more successful performance of much more integrated and highly comprehensive monitoring of ground subsidence over areas of various sizes and complexity.
